# Dynamics of surface of lipid membranes: theoretical considerations and the ESR experiment

**DOI:** 10.1007/s00249-016-1172-8

**Published:** 2016-09-17

**Authors:** Dariusz Man, Ryszard Olchawa

**Affiliations:** Institute of Physics, Opole University, Oleska 48, 45-052 Opole, Poland

**Keywords:** Lipid membrane, Membrane fluidity, Monte Carlo simulation, ESR probe

## Abstract

The effect of the surface layer of model membranes on their physical properties was discussed in this paper. The research involved a physical ESR experiment with the use of spin probes and computer simulation based on the Monte Carlo technique. Liposomes formed during the process of sonication of lecithin were scanned in an ESR spectrometer. The membrane surface layer model, represented by the system of electric dipoles arranged in rectangular or hexagonal matrices, was studied. The final states of computer simulations were presented as textures. It was found that in the gel phase some ordered domain structures are formed, while in the liquid–crystal phase we got complex textures comprising a plurality of gaps. The process of forming domain structures during the changing of the temperature and the phase transitions taking place in a dipole system as a function of dipole mobility (*k*-parameter) was presented. The results obtained imply that the head groups (represented by electric dipoles in the computer model) of the surface layer play a key role in membranes, affecting the properties of the entire membrane, which is particularly essential for transport processes. It also modified the characteristics of the membrane gel-liquid crystalline transition phase.

## Introduction

Biological membranes play a fundamental role in the functioning of all living organisms. They have a complex and dynamic structure (Singer and Nicolson 1972) whose elements are constantly moving (McConnell 1976; Sackmann 1978). Because of the considerable complexity of these systems, developing a mathematical model covering all interactions is very difficult; therefore the proposed models of membranes concentrate on selected groups of physical interactions. The basic structure of a biological membrane is a lipid bilayer, which constitutes its core. It serves as a foundation to which other elements determining individual properties of the membrane are attached. Learning about mechanisms affecting the properties of the lipid bilayer (the structural core of membranes) will allow for a better understanding of processes occurring in the actual biological membrane. Liposomes provide a good model for studying the physical properties of real biological membranes. Both their molecular composition and geometric dimension can be precisely controlled, meaning that the studied objects are well defined, whilst at the same time, being complex enough to reflect the properties of natural biological membranes. In order to understand better the processes taking place in the lipid bilayer, in parallel to the physical experiment, other studies have focused on using mathematical models. One of the earliest was the model presented in 1980 and 1998 by Pink et al. 1998 who assumed that the area determining the dynamic properties of the membrane is their hydrophobic interior, comprising long hydrocarbon chains. The properties of the surface layer were not taken into consideration. Because of the specificity of the object, the computer-based techniques have become an extraordinarily helpful tool in imaging and providing a better understanding of the processes occurring in the membranes. The computer-based models can be divided into two groups: deterministic (e.g., implemented in biomolecular codes: GROMACS, CHARMM), which are based on numerical solutions of movement equations of a system, e.g., molecular dynamic simulations, which illustrate time-dependent phenomena (Pasenkiewicz-Gierula et al. 1999; Lopez et al. 2002; Berendsen and Tieleman 2014), and the Monte Carlo method, which in its broadest meaning introduces an element of randomness into the algorithms of calculations. The Monte Carlo method is most often applied in those cases where the deterministic methods are not appropriate because of the large degree of freedom in a given system. One example of the application of this method is when generating a large set of configurations of the examined system, which meet preset physical conditions (of an ensemble) and then calculating the mean of this group of states. This model, applied to lipid membranes, can be found in the following studies on: gel-fluid transitions (Mouritsen et al. 1983; Kubica 1997), acyl density fluctuations (Ipsen et al. 1990), the binding energy of a lipid surface (Man et al. 2004, 2007, 2010, 2013a), the effects of cholesterol (Ipsen et al. 1989, 1990), influence of membrane modifiers on membrane properties (Jorgensen et al. 1991a), partitioning of membrane intruders (Jorgensen et al. 1991b), membrane heterogeneity, lateral distribution of proteins in membranes, and on membrane electroporation (Kotulska et al. 2007). The Monte Carlo method enables one also to simulate systems in which not all interactions are known, but can be given stochastic characteristics (e.g., Browns dynamics). Just this aspect of the Monte Carlo method was used in the presented computer simulation to model the effect of the inner membrane layer on the processes occurring on its surface layer. The advantage of this approach in comparison to the deterministic methods is the possibility to overcome the problem of local minima of the potential energy. The potential energy of the rotating dipoles has a lot of local minima. As the force always points according to the gradient, the system state can be trapped in a local minimum until the end of the simulation. Adding some random forces (seen as the chaotic influence of the environment related to the temperature) allows the simulated system to jump out of the local minimum and finally achieve the global one. This problem is shown in this article. The random forces also minimize the effect of the truncation of electrostatic interaction (Karttunen et al. 2008). The best insight can be gathered through a combination of experimental and theoretical works, as in the presented material, where the authors have combined a physical experiment where liposome membranes were studied (using an electron spin resonance (ESR) technique), with computer simulation modeling of the surface layer of the membrane. The ESR method was chosen because it allows one to perform analysis of subtle differences in membrane fluidity taking place in its various regions (in cross section), resulting from various physicochemical stimuli, e.g., temperature changes, and the effects of types and concentrations of admixtures (modifiers) placed in the membrane (Man 2008; Man et al. 2009, 2010, 2013a, b). The authors noted that under the impact of certain modifiers and with the passage of time, spectroscopic parameters of spin probes penetrating the polar (surface) layer of the membrane displayed greater change dynamics than the probes placed in the hydrophobic part (middle part of bilayer). Such results suggest that the processes of admixture distribution in the membrane depends to a great extent on the properties of the surface layer a water/lipid interphase. The objective of this study is to attempt to explain the impact the surface layer of membranes has on their structural properties, as well as studying the relationships between the mobility of molecules and the appearance of domain structures composed of head groups on the membrane surface. The computer model is designed to answer the question of whether there is a border value of the mobility of head groups (electric dipoles) that determines the phase transition in this region of the membrane. The authors are of the opinion that such a parameter exists, as suggested by the results obtained in the work by Man et al. (2010). Distinct differences were observed in the arrangement of electric dipoles associated with their mobility. Besides, a strong influence of impurities present at the membrane surface on the nature of the thermotropic phase transition was detected and observed inside the membrane. This article is focused on the investigation of electric interactions in the surface layer by treating the molecules of this layer as electric dipoles. Finding a relationship between the mobility of polar heads and the rate of creation of dynamic defects (as postulated in the work by Iwkow and Bieriestowskij 1982) enables designing membranes with specific physical properties for a given set of physical environmental conditions, such as pH, temperature, degree of hydration, and the like. This knowledge would be very valuable for developing medicines based on liposomes or nanotechnology using liposomes as intelligent transporters. The active substances (e.g., drugs) contained in liposomes, once introduced into the body, can be released in a controlled manner, thanks to the possibility of designing the transient pore size in the hydrophilic layer.

## Materials and methods

### ESR experiment

**Fig. 1:**
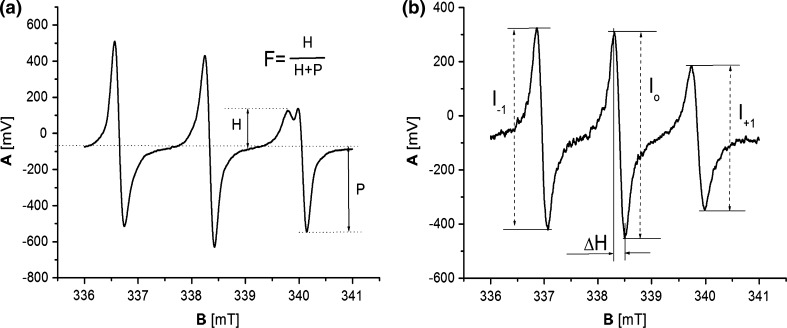
ESR spectra of spin probes placed in the liposome membrane. **a** TEMPO probe and spectroscopic partition parameter (*F*). **b** 16-DOXYL-stearic acid methyl ester probe and parameter (*τ*), rotation correlation time $$\tau = 5. 9 5\cdot {\text{H}}\left( {\left( {{\text{I}}_{0} /{\text{I}}_{ + 1} } \right)^{\raise.5ex\hbox{$\scriptstyle 1$}\kern-.1em/ \kern-.15em\lower.25ex\hbox{$\scriptstyle 2$} } + \left( {{\text{I}}_{0} /{\text{I}}_{ - 1} } \right)^{\raise.5ex\hbox{$\scriptstyle 1$}\kern-.1em/ \kern-.15em\lower.25ex\hbox{$\scriptstyle 2$} } - 2} \right) 10^{ - 10} \left[ {\text{s}} \right]$$

**Fig. 2:**
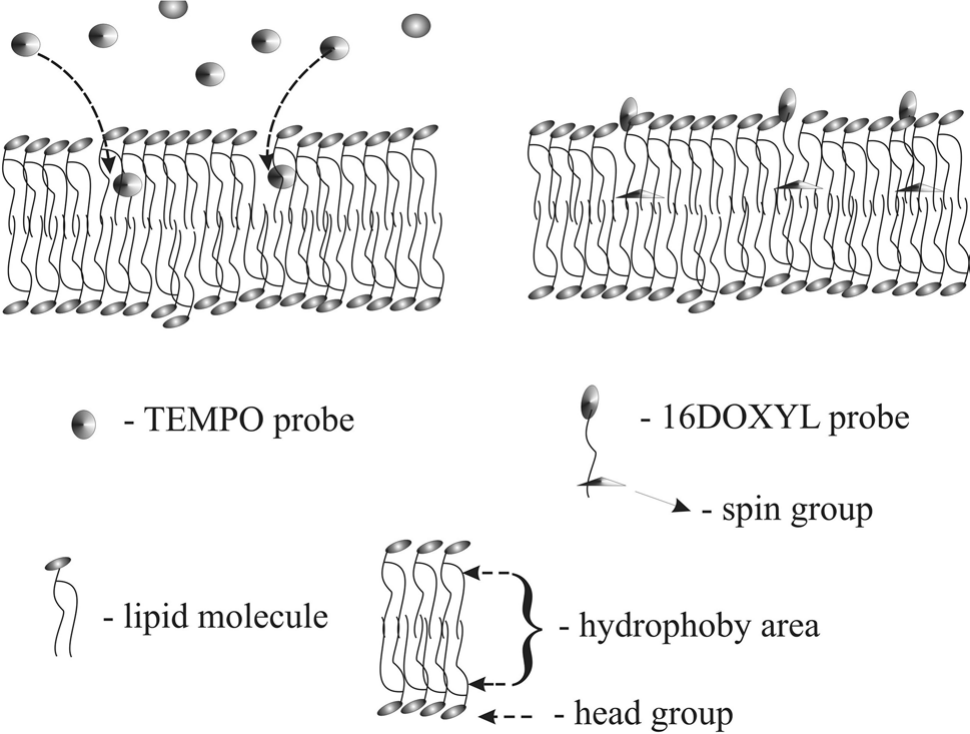
Scheme showing the locations of probes in the lipid bilayer of liposome

The spin probes, 2-ethyl-2-(15-methoxy-15-oxopentadecyl)-4, 4-dimethyl-3-oxazolidinyloxy (16-DOXYL-stearic acid methyl ester), and 2,2,6,6-tetra–methylpiperidine-1-oxyl (TEMPO) as well as the dipalmitoylphosphatidylcholine (DPPC) and Egg Yolk Lecithin (EYL), were supplied by Sigma-Aldrich Sp. z o.o. Poznań, Poland. The liposomes were obtained through sonication of DPPC and EYL lecithins in distilled water, using an ultrasonic disintegrator (UD-20; Techpan, Warsaw, Poland). The total sonication time for a single sample of a volume of 1.5 ml was 4 min and the process consisted of a sequence of 30 s sonication cycles followed by 30 s of cooling. The DPPC and EYL concentrations in the sample were 40 mM. After the sonication process, the water dispersion of liposomes was divided into two parts and the TEMPO and 16DOXYL spin probes, in concentrations of 1000 ppm relative to lecithin (DPPC or EYL), were added to these, respectively. Next, the samples containing liposomes and probes were shaken for 10 min, and then left for 15 min in order to stabilize in 24 °C. Next, the samples were placed in a measuring chamber of an ESR spectrophotometer in thin glass capillaries and scanned. From the analysis of spectroscopic images of spin probes (Fig. 1), information regarding the fluidity of membranes in their surroundings was obtained: for the surface layers for the TEMPO probe, and for the inner layer for the 16DOXYL probe. The places where probes were located are presented in Fig. 2. From the ESR spectrum of the TEMPO probe the spectroscopic partition parameter (*F*), reflecting the probe distribution between the membrane and its ambient, was determined. A measure of the parameter *F* is the ratio of the high-field line amplitude in the ESR spectrum of the probe, dissolved in the water ambient (*P*) to the amplitude of the low-field line (*H*) in the lipid environment (Fig. 1a), The value of *F*, among other parameters, is connected with the membrane fluidity (Shimshick and McConnell 1973). From the spectrum of the 16-DOXYL-stearic acid probe, the spectroscopic parameter *τ* was determined. Its value depends, among other things, on the degree of the membrane fluidity and is greater the more rigid (better ordered) the ambient of the probe (Hemminga 1983) is. In the case of an isotropic environment, *τ* is the rotation correlation time of the probe (Fig. 1b).

### Computer-based model

**Fig. 3:**
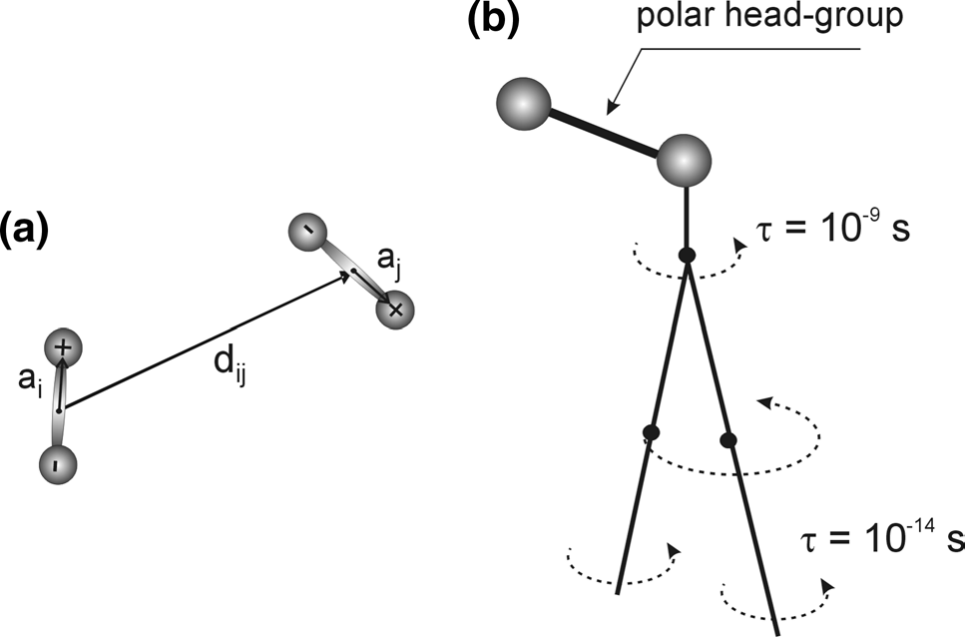
**a** The parameterization of the dipoles. **b** A schematic illustration of the dynamics of phospholipid molecules

In order to better understand the processes occurring in the surface of a membrane, a computer-based model was developed. The computer model was comprised of a system of N electric dipoles, which can rotate around its own axis perpendicularly to the surface of the membrane, as well as move over the plane of the membrane. The total energy of the surface layer system (Hamiltonian) has the following form:

1 $$H = \sum\limits_{(i)} {\frac{{p_{i}^{2} }}{2m} + } \sum\limits_{(i)} {\frac{{L_{i}^{2} }}{2I} + \sum\limits_{(i < j)} {U_{ij} } } ,$$

where *m* is the mass of the dipole, and *I* is its moment of inertia. The first two terms describe the kinetic energy associated with translational and rotational movement of dipoles (Fig. 3). The third part represents Coulomb and Lennard–Jones interactions between all dipoles.

2 $$U_{ij} = \frac{{e^{2} }}{{4\pi \varepsilon_{0} \varepsilon_{r} }}\left( {\frac{1}{{\left| {\overrightarrow {d}_{ij} + \overrightarrow {{a_{j} }} - \overrightarrow {{a_{i} }} } \right|}} - \frac{1}{{\left| {\overrightarrow {{d_{ij} }} - \overrightarrow {{a_{j} }} - \overrightarrow {a}_{i} } \right|}} + \frac{1}{{\left| {\overrightarrow {{d_{ij} }} - \overrightarrow {{a_{j} }} + \overrightarrow {{a_{i} }} } \right|}} - \frac{1}{{\left| {\overrightarrow {{d_{ij} }} + \overrightarrow {{a_{j} }} + \overrightarrow {{a_{i} }} } \right|}}} \right) + 4\varepsilon \left[ {\left( {\frac{\sigma }{{\left| {\overrightarrow {d}_{ij} } \right|}}} \right)^{12} - \left( {\frac{\sigma }{{\left| {\overrightarrow {d}_{ij} } \right|}}} \right)^{6} } \right].$$

The meanings of vectors **a** and **d** are described in Fig. 3a.

Therefore, the system has three degrees of freedom, one associated with a rotational motion, and two associated with a translational motion. In a real membrane, these dipoles represent heads of chains whose motion is markedly restricted by the chains connected with the heads. In particular, the presence of these chains restricts the possibility of the heads (dipoles) from coming close to each other. It is obvious that the dynamics of the chains connected with the heads greatly affects the dynamics of the heads. In our model, we have assumed that the impact is purely stochastic and uncorrelated, which is, obviously, a simplification. Our model can be viewed as a system of dipoles placed rigidly on round corks, floating on a lipid sea. This model is designed to determine the critical relation between two competing processes: rotational motion and translational motion, which qualitatively changes the structure of the membrane surface. In the course of our studies, it was found that the dipole system displaying significant rotational mobility in relation to translational one leads to end-phases different from that in the opposite situation. In the model used, the parameter defining the relation between these degrees of freedom is parameter *k* (defined later in the text), which determines the ratio between the mean value of displacement of the dipole on the membrane surface and the value of the angle of dipole rotation in a single simulation step. The increasing value of this parameter corresponds to greater mobility in the translational motion. The starting point for this definition is the principle of equipartition of energy, which states that the mean kinetic energy in rotational motion is the same as the mean kinetic energy in the translational motion along each axis (*x* and *y*):

3 $$\frac{{\left\langle {p_{{x_{i} }}^{2} } \right\rangle }}{2m} = \frac{{\left\langle {p_{{y_{i} }}^{2} } \right\rangle }}{2m} = \frac{{\left\langle {L_{i}^{2} } \right\rangle }}{2I}.$$

Equation (3) shows the relation between the translational and rotational motion:

4 $$\left\langle {L_{i}^{2} } \right\rangle = \frac{{I\left\langle {p_{{_{i} }}^{2} } \right\rangle }}{2m}.$$

Let *t* denote a simulation time step, then the mean value of displacement $\left\langle {{\text{d}}x} \right\rangle$, $\left\langle {{\text{d}}y} \right\rangle$ and rotation $\,\left\langle {{\text{d}}\alpha } \right\rangle$ in a single time step could be written in the following form:

5 $$\left\langle {{\text{d}}x} \right\rangle = \frac{{\left\langle {p_{x} } \right\rangle }}{m}\,t,\,\,\left\langle {dy} \right\rangle = \frac{{\left\langle {p_{y} } \right\rangle }}{m}\,t,\,\left\langle {{\text{d}}\alpha } \right\rangle = \frac{\left\langle L \right\rangle }{I}\,t.$$

Using a classic relationship for mean values:

6 $$\left\langle {\left( {\delta A} \right)^{2} } \right\rangle = \left\langle {A^{2} } \right\rangle - \left\langle A \right\rangle^{2} ,$$

we can write the relation (3) between the mean values of momentum and angular momentum in the following form:

7 $$\left\langle L \right\rangle^{2} + \left\langle {\left( {\delta L} \right)^{2} } \right\rangle = \frac{I}{2m}\left( {\left\langle p \right\rangle^{2} + \left\langle {\left( {\delta p} \right)^{2} } \right\rangle } \right).$$

The above consideration is only of a qualitative nature, as it does not take into account the effect of the lipid membrane chains upon the dynamics of the studied dipoles moving on the surface of the membrane. It is not possible to consider formally the impact the aquatic environment (WeiXin et al. 2016) as well as the interaction between the heads of lipids (dipoles) and chains because neither the dynamics of the chains nor their effect on the lipid heads are sufficiently well known. In this study, we assume that these interactions are of a stochastic nature, and may affect both degrees of freedom to a different extent. This difference effects results in a mean range of rotational motion and can be greater or smaller than implied by the relationships (4) and (5). In a specific case, the lipid chains can prevent the entire relocation of dipole axes, as such membrane models have been examined in earlier works (Mouritsen et al. 1983; Kubica 1997; Man et al. 2010, 2013a). Similar to the article of Man et al. (2010), we introduce a dimensionless parameter *k*, describing the mutual mean range in the translational and rotational motions:

8 $$k = \frac{\pi }{l}\,\frac{{\left\langle {{\text{d}}x} \right\rangle }}{{\left\langle {{\text{d}}\alpha } \right\rangle }},$$

where *l* is the length of the dipole. Low values of this parameter practically freeze the translational movement, and the end-phase obtained has the structure of domains determined by dipole orientation, whereas (as it was observed during simulations) the high values of this parameter lead to a different end-phase with a strongly linear (chain) aggregation of the dipoles. Considering the experimental observations, we can associate this parameter with the viscosity of the membrane. The objective of simulation is to determine the threshold value of this parameter, which divides these two areas of states. Each simulation is carried out for the set parameter *k* and temperature. The simulation begins from a determination of the initial state of the system through random distribution of dipoles or from reading in a set initial state. During the simulation, a sequence of system states in the subsequent simulation steps (Markov chain) is generated (Rapaport 1996; Varlet 1968; Hermann 1986; Thijssen 1999). The algorithm of simulation is implementation of the Metropolis algorithm. It can be roughly described as a random walk in the space of states with a strong orientation towards the direction of minimum total energy of the dipole system interactions. In each step in which a drawn dipole is rotated for a random angle, a random relocation is made, thereby retaining the relationship (8). Obviously, this change of state leads to a change in the total potential energy of the system:

9 $$E_{\text{p}} = \sum\limits_{(i < j)} {U_{ij} } ,$$

by value $$\Delta E_{\text{p}}$$.

In accordance with the Metropolis algorithm procedure, such a new state of the system automatically becomes the current system if $\Delta E_{\text{p}} \le 0$, whereas in the opposite case this state can be accepted in accordance with the probability:

10 $$w = \exp \left( { - \frac{{\Delta E_{\text{p}} }}{{k_{B} T}}} \right),$$

where $k_{B}$ is the Boltzmann constant, and *T* is the temperature. In the case of rejection, the previous state becomes the current system state. In the initial phase, we observe a rapid decrease in the energy of interaction, and after a certain time, the system reaches a state of equilibrium, this energy then stabilizes and oscillates around a certain mean value. The configuration of states so obtained is determined by the given parameter *k* and temperature *T*.

## Results and discussion

### ESR experiment

#### Thermotropic phase transitions in pure and modified DPPC membranes

In order to test the effect of the surface layer on the dynamic properties of the entire membrane, the phase transition: gel-liquid crystal was examined and compared for liposomes made from DPPC, without a modifier, and with a modifier placed on the surface of the membrane. A technique of spin probes based on the nitroxyl group a sustainable paramagnetic center was selected for the experiment. The 16DOXYL probe, which locates its active spin in the center of the lipid bilayer (Fig. 2), was selected for the experiment. The speed of spin rotation around the axis of symmetry of the probe depends on ambient viscosity. The analysis of the rotational spectrum of the probe (Fig. 1) provided information about its immediate surroundings, through determining the parameter *τψ*-time of the rotational correlation.

11 $$\tau = 5.95 \cdot \Delta {\text{Ho}} \cdot \left( {\sqrt {\frac{{I_{0} }}{{I_{ + 1} }}} + \sqrt {\frac{{I_{0} }}{{I_{ - 1} }}} - 2 } \right) \cdot 10^{ - 10} S,$$

where parameters *I*_0_, *I*_−1_ and *I*_*+*1_ are explained in Fig. 1.

**Fig. 4:**
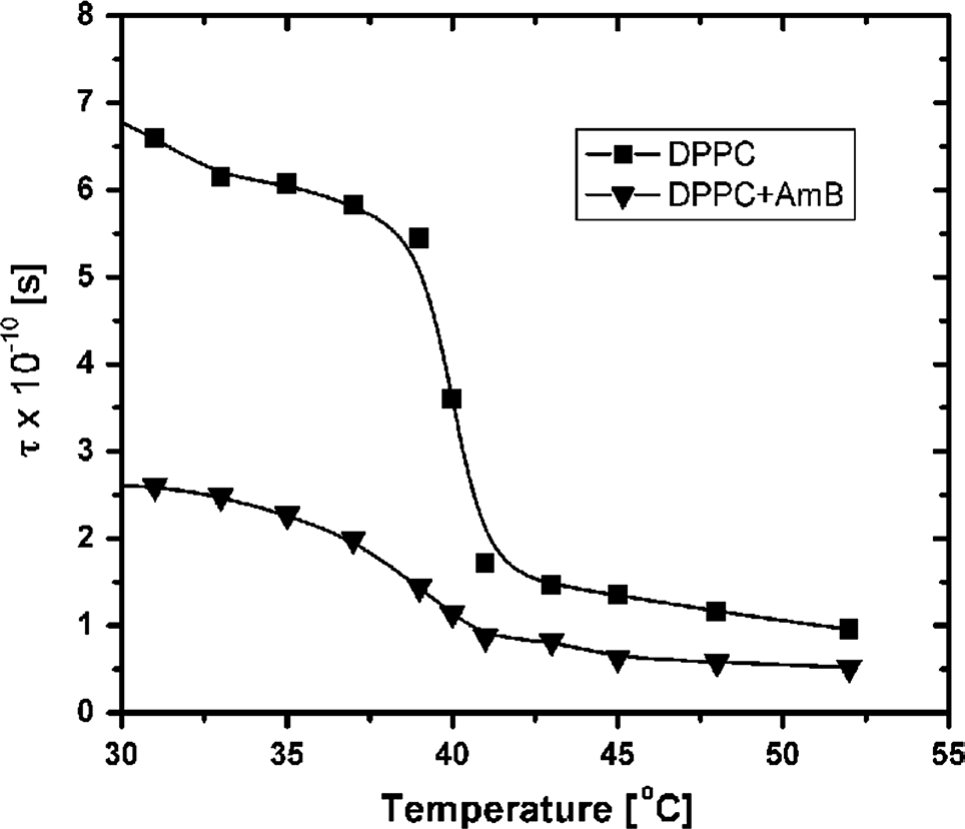
Graph illustrating the phase transitions gel-liquid crystal for the membrane without a modifier (*squares*), and with a modifier (Amphotericin B—0.5 %) placed on the surface of the membrane (*triangles*)

Figure 4 shows that in the case of liposomes membranes made of pure DPPC, there was a rapid, evident phase transition for a temperature of 40 °C. Such a result is known from the publications and is only a reference point for the next experiment. In the case of the second curve (denoted by triangles, Fig. 4), for comparison, the DPPC was modified by the AmB molecules. The AmB were used because their behavior in contact with the membrane was already well described (Gruszecki et al. 2003; Hereć et al. 2005). It is known (Man and Olchawa 2013) that for a 0.5 % concentration, the molecules are placed on the surface layer (and do not penetrate the bilayer). As one can see, in this case the phase transition is considerable less marked. This means that in the case of membranes made of the same material there has been a radical change in the properties of the entire membrane; the probe registered the changes in the ambient inside the bilayer (Fig. 2). Coefficient *τψ* time of rotational correlation of the 16DOXYL was determined in accordance with formula (11).

#### Effect of time

**Fig. 5:**
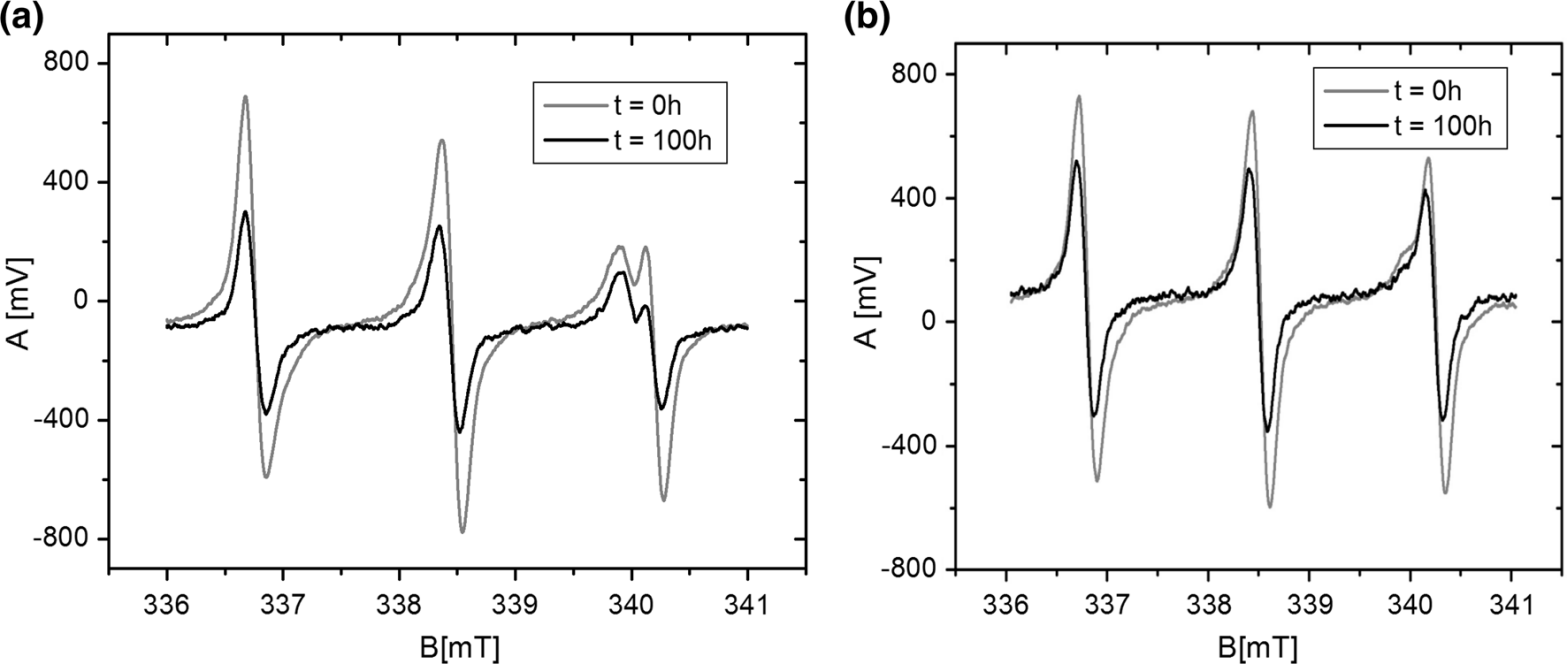
Spectroscopic lines of the TEMPO probe recorded for the liposome membranes in the liquid-crystalline phase (**a**), and gel phase (**b**), at the beginning of the measurements and after 100 h

In the second experiment, the effect of time on the process of penetrating the membrane in a liquid–crystalline state and in the gel phase, by a small amphiphilic molecule, was examined. For this purpose, liposomes were made from EYL and DPPC. The EYL membranes in the temperature of the experiment (24 °C) were in the liquid–crystal state, whereas DPPC membranes under the same temperature were in the gel phase. The TEMPO probe used as a marker displays high mobility and dissolves both in aquatic and lipid environments. The probe was first introduced into the aquatic environment of liposome dispersion, which means that it had, on its way, to pass through the surface layer before reaching the inner parts of the membrane. Because of the different polarity, the high-field line in the ESR spectrum of this probe, in the lipid environment, is located a bit earlier than in the aquatic environment. This permits analyses of the division of the probe between these two environments. Figure 5 shows that in the case of liposomes in the liquid–crystalline phase, the quantity of the probe penetrating the lipid environment increased over time. After *t* = 100 h from the beginning of the experiment, a substantial quantity of the probe dissolved in the membrane, which signifies a significant increase in the peak (closer to the central line the bold curve) compared to the state at *t* = 0 h. In the case of liposomes made of DPPC whose membranes were in gel phase, no such changes were found. This means that in this case the probe was not able to penetrate the surface layer of the membrane.

These results indicate the importance of the surface layer in transport phenomena. We think that in the liquid–crystal phase, on the surface membrane, the gaps were formed, with an area of several nm^2^ (Man et al. 2010). Because of this, the effective surface area of the interaction of interphase with environment increases. So we have to deal with two factors contributing to the process of penetration of the membrane interior by molecules from an aqueous environment (probe TEMPO): the increase of the effective surface area and reduce the viscosity of the medium. In the case of membrane phase gel, polar head groups form the structure of the domain, uniformly filling the plane (no gaps), which results in a reduction in the effective surface area of the interface between membrane and aquatic environment. The particles dissolved in the liposome environment (probe TEMPO) have limited access to the interior of the membrane, what showed the results of ESR.

### Simulation results

#### Membrane in the gel phase

**Fig. 6:**
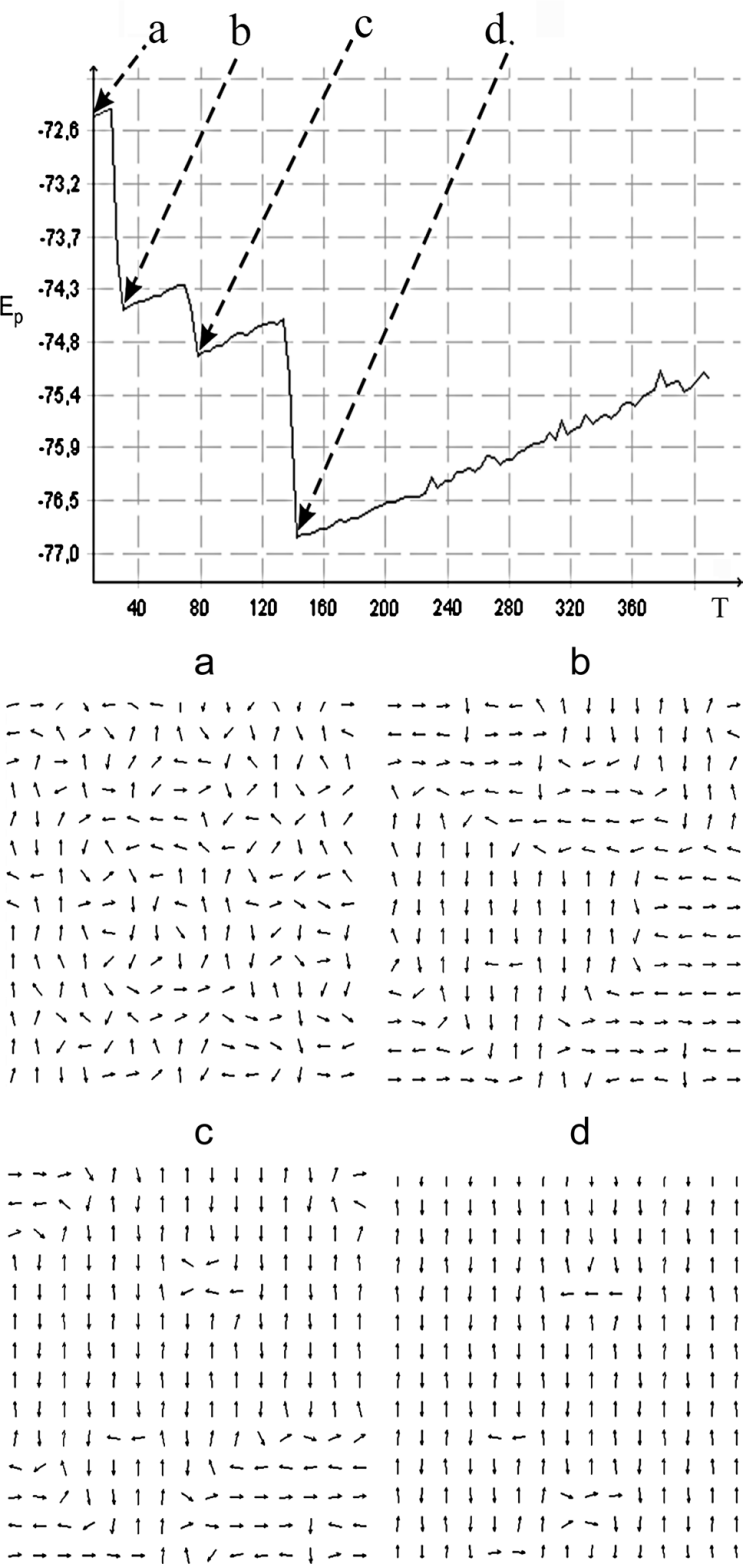
Relationship between potential energy *E*p of the system of 15 × 15 dipoles and temperature *T* (*K*). The abrupt changes in energy correspond to the changes in distribution of electric dipoles (domains), from the state *a* (disordered) via intermediate states *b* and *c*, to the (approximately) anti-parallel structure *d*

The objective of this simulation was to check the behavior displayed by the surface layer of the membrane in the gel phase under the conditions of rising temperature. Dipoles have freedom only to rotate, as their centers are limited in translational mobility and arranged in a rectangular matrix of 15 × 15 dipoles. Figure 6 presents the changes in the binding energy of the system of dipoles caused by a rise in temperature. At the beginning of the simulation, the dipole orientations were set with the use of a random number generator. The number of steps in the simulation was set so as to allow the system to reach a state of equilibrium for particular temperatures. The dipole dimensions were set at *l* = 0.5 nm, in accordance with the dimensions given in the work by Stigter et al. (1992) for phosphatidylcholine. The distances between the centers of the dipoles were set at 2*l*. The emerging structures assumed the form of domains whose arrangements changed in steps.

Figure 6 illustrates how thermal energy (*kT*) causes vibration dipoles, and precipitates them from metastable states (domain structures) aiming to set the dipoles to the minimum binding energy system (antiparallel structure for the whole area). This visualization shows what can happen on the membrane surface, which is in a gel. Interestingly, the simulation shows that even at very low temperatures, the dynamic processes on the surface of the membrane can occur. Perhaps such knowledge will be useful for understanding the processes occurring in biological membranes at extremely low temperatures (cells frozen in liquid nitrogen).

#### Transition of the membrane from a gel phase to the liquid–crystalline phase

**Fig. 7:**
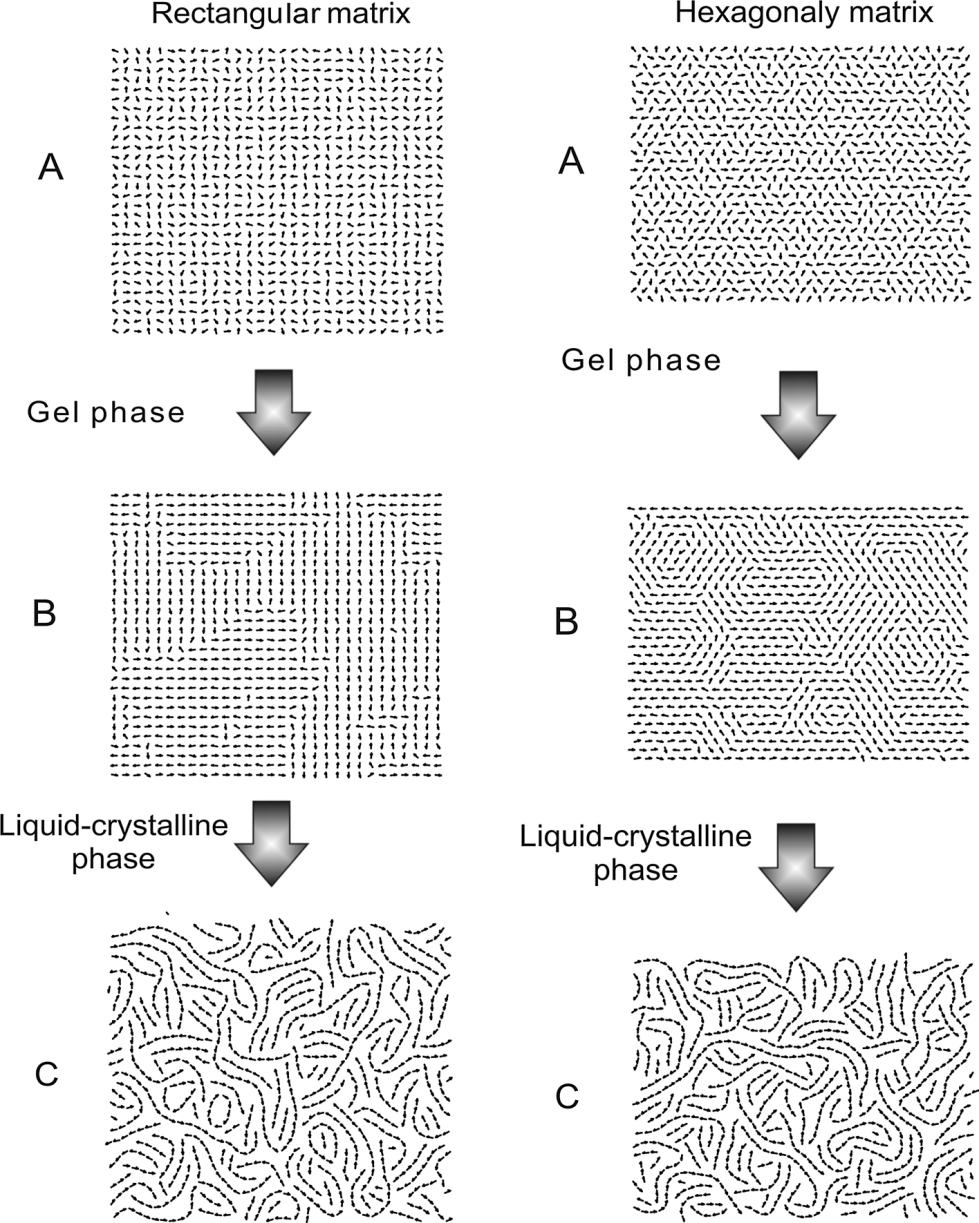
Images of selected bitmaps for rectangular and hexagonal matrices, 30 × 30 dipoles: **a** beginning of simulation, **b** gel phase for *k* = 0.1, **c** liquid–crystalline phase for *k* = 0.6

**Fig. 8:**
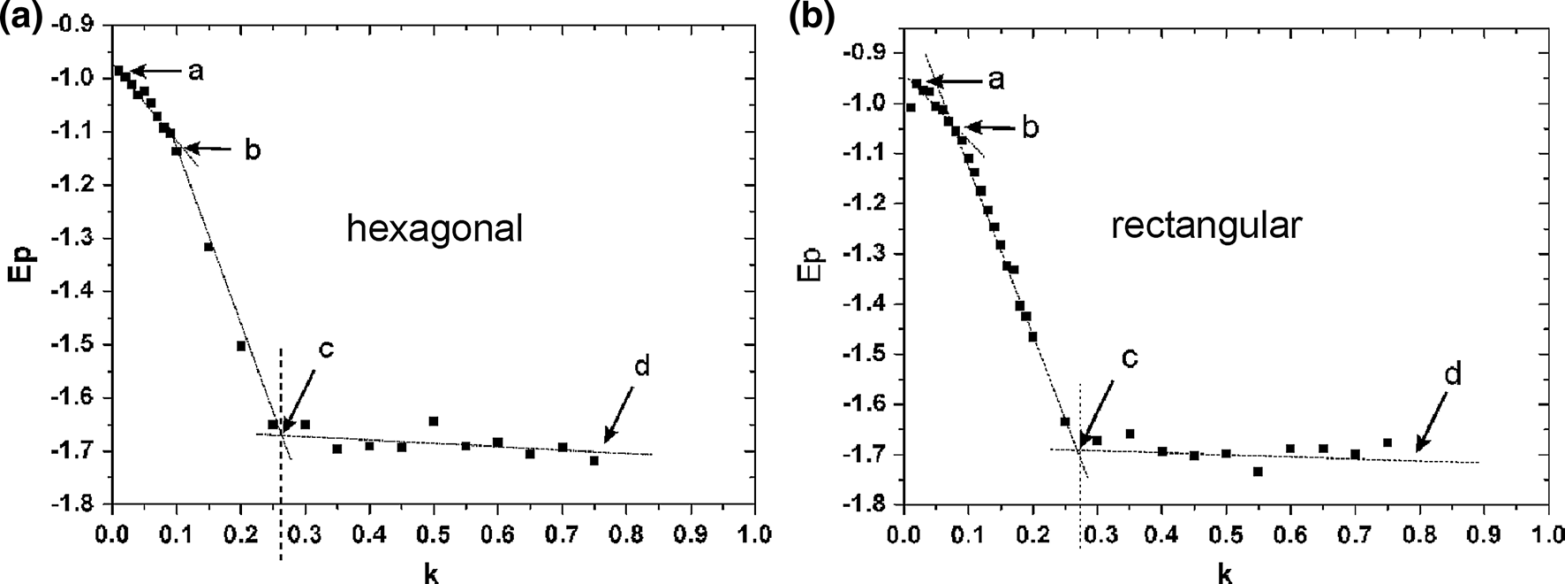
The relationship between the relative binding energy *E*p for a 30 × 30 dipoles system of the *hexagonal* (**a**) and *rectangular* matrices (**b**), and the coefficient *k*. Each* point* corresponds to the final state of a single simulation with fixed value of the *k* parameter. The section *ab* corresponds to the gel phase, *bc* phase transition, *cd* liquid–crystal phase

**Fig. 9:**
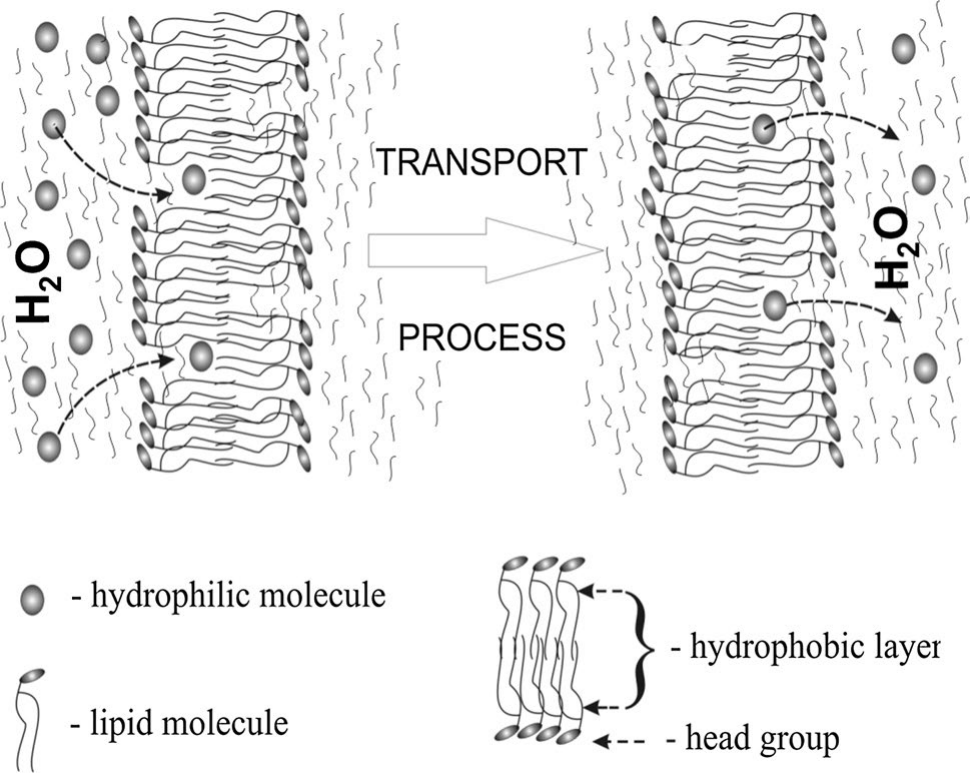
A probable mechanism of the transport of hydrophilic molecules, across the hydrophobic area of the lipid membrane

The objective of this simulation was to check the behavior displayed by the surface layer of the membrane, with the increasing freedom of the translational motion, which in real conditions corresponds to the increase in fluidity of the membrane. Dipoles have freedom of rotation and translational motion, and the range of the latter is regulated by the parameter *k*. The value of the parameter *k* near 0 corresponds to the situation when the membrane is in the gel state (dipoles are limited in rotational mobility). The increase in the value of parameter *k* corresponds to the increase in mobility; for larger *k* we have the state of dissolution (disintegration) of the membrane. In order to fully illustrate this phenomenon, the simulations were conducted for two matrices comprising 30 × 30 dipoles of rectangular and hexagonal symmetry Figs. 7a, b, c (hexagonal symmetry was discussed in the work by Raudino and Mauzerall 1986). Position A represents the initial state of the simulation where the dipole orientations were set with the use of a random number generator, (which corresponds to the first measuring point in Fig. 8). Position B shows the system for *k* = 0.1, which corresponds to the gel phase, and matches the area marked in Fig. 8 as section ab. Characteristic domain structures appeared (typical for the gel phase) without evident gaps in the interphase. Position C shows the system after the main phase transition for *k* = 0.6, which matches the area marked in Fig. 8 as section cd. The distribution of dipoles for both matrices resemble each other similar textures emerge which, in the cases of great molecule mobility, is a logical result. A number of gaps can be noted in the distribution of dipoles, which in real conditions corresponds to the defects in the surface layer of the membrane. These defects can be responsible for penetration of molecules from one side of the membrane to the other (Fig. 9). The section denoted bc in Fig. 8 represents the transitional area, where the domain structures undergo gradual disintegration up to the point where textures characteristic for the image shown in Fig. 7c are formed.

The results indicate the occurrence of abrupt changes of the binding energy (*E*p) of the dipoles, as a function of motility (parameter *k*). With regard to the real biological membranes, such a result can be interpreted as the existence of a phase transition in the area of polar head groups, for a particular level of their mobility (membrane fluidity). This result may indicate a strong influence on the interphase region on the properties of the entire membranes. Such a behavior of the system under study is the result of the game between Coulomb and L–J interactions. In the case of the gel phase the L–J interaction dominates, while in liquid–crystal phase the Coulomb interaction is much stronger.

## Conclusions

Results obtained in this study confirmed the existence of a critical value of the parameter *k* (*kc*), which separates the two fundamentally different types of final states of the simulation. Simulations for *k* < *kc* led to the final states corresponding to membranes in gel phase, whereas for *k* > *kc* to membranes in the liquid–crystal phase.

Moreover:

1. Changes in the surface layer of the membrane significantly affect the properties of the entire membrane, changing the characteristics of the phase transition (Fig. 4).
2. The state of the surface layer affects the process of diffusion of molecules between the membrane and aquatic environment (Fig. 5).
3. In the gel phase, the polar heads of phospholipid molecules form domain structures which, with the rise in energy, take up approximately anti-parallel positions (Fig. 6).
4. In the liquid-crystal phase, the gaps emerge on the membranes surface (Fig. 7c), which enable penetration of molecules through the surface interphase of the membrane to its hydrophobic interior (Fig. 9). A similar process associated with defects in the membrane surface leading to metastable water pores was described in Gurtovenko et al. (2010).

**Fig. 10:**
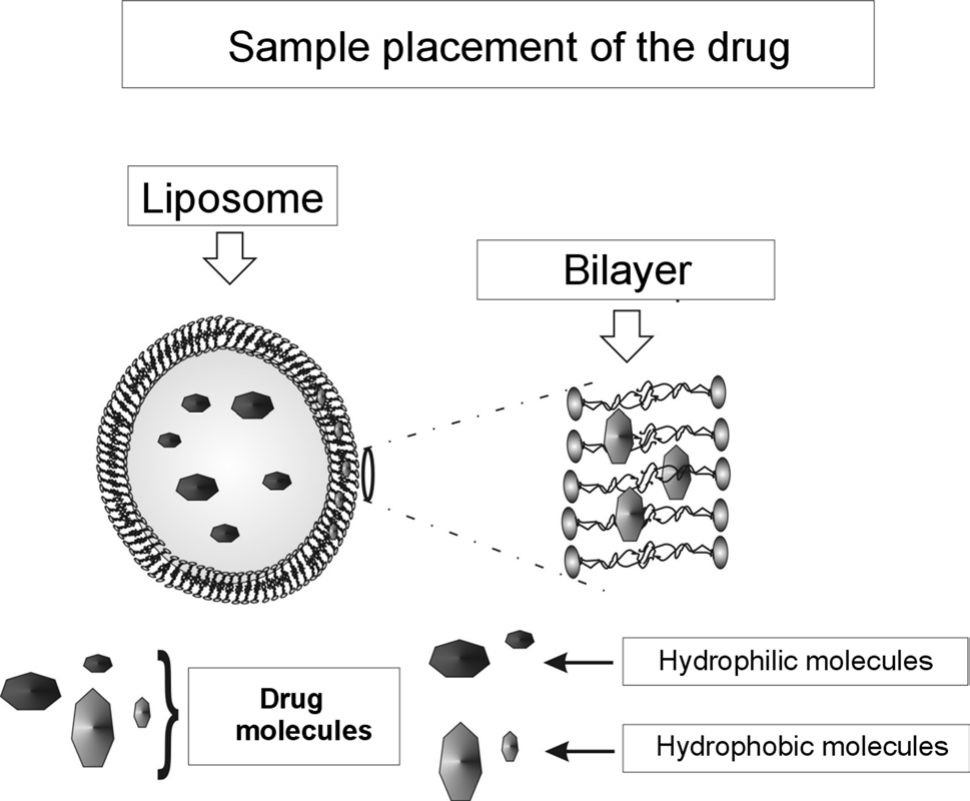
Sample placement of the drug in the liposomes

The presented results can explain the phenomenon of transportation of hydrophilic molecules (e.g., transport of ions) through lipid membranes (biological membranes). This model explains, in a simple manner, the phenomenon involving the transport of Na^+^, K^+^, Ca^++^, and Cl^−^ ions through the hydrophobic interior of the lipid bilayer. Learning and gaining control of the mechanism regulating the size of gaps in the surface layer will enable one to develop new technologies (nanotechnologies) using liposomes as intelligent transporters; namely to provide doses of medicine inside an organism in a specific place and time. Drugs with hydrophobic and hydrophilic should be placed in liposomes: hydrophobic molecules in lipid bilayer and the hydrophilic inside liposome Fig. 10. Thus, prepared drugs can be introduced into the patient and then released through the pores, or by fusion of the membranes of tissues. Adjusting the pore size of the membrane enables control of the rate of diffusion of the drug into the body.

This can be of great importance in the production of new-generation patient-friendly medicines.
